# Incident arterial vascular events in a cohort of Puerto Ricans with rheumatoid arthritis

**DOI:** 10.1177/2050312120958844

**Published:** 2020-09-15

**Authors:** Ariana González-Meléndez, Ruth M Fred-Jiménez, Mariangelí Arroyo-Ávila, Leyda Díaz-Correa, Naydi Pérez-Ríos, Noelia Rodríguez, Grissel Ríos, Luis M Vilá

**Affiliations:** 1 Division of Rheumatology, Department of Medicine, University of Puerto Rico Medical Sciences Campus, San Juan, Puerto Rico; 2 Puerto Rico Clinical and Translational Research Center, University of Puerto Rico Medical Sciences Campus, San Juan, Puerto Rico

**Keywords:** Rheumatoid arthritis, arterial vascular events, clinical outcomes, Hispanics, Puerto Ricans

## Abstract

**Objective:**

The increased morbidity and mortality associated with cardiovascular events in patients with rheumatoid arthritis has been linked to traditional and nontraditional factors. However, these factors vary among different ethnicities. Few studies have described these features in Hispanic populations. Thus, we determined the clinical correlates of arterial vascular events in Hispanics from Puerto Rico.

**Methods:**

A cross-sectional study was performed in a cohort of 405 Puerto Ricans with rheumatoid arthritis. Demographic parameters, health-related behaviors, clinical manifestations, disease activity (per Disease Activity Score 28), functional status (per Health Assessment Questionnaire), comorbidities, and pharmacotherapy were compared in patients with and without incident arterial vascular events. The latter was defined as the occurrence of myocardial infarction, angina pectoris, vascular procedures for coronary artery disease, stroke, or peripheral artery disease. Study groups were analyzed using bivariate and multivariate analyses.

**Results:**

Of the total study population, 87.2% were woman. The mean age at study visit was 56.1 ± 13.9 years, and the mean disease duration was 15.0 ± 13.2 years. Arterial vascular events occurred in 43 patients (10.6%). In the multivariate analysis adjusted for age and sex, arterial hypertension, dyslipidemia, metabolic syndrome, extra-articular manifestations, higher Health Assessment Questionnaire score, and number of hospitalizations were associated with arterial cardiovascular events.

**Conclusion:**

In this cohort of Puerto Ricans with rheumatoid arthritis, traditional and nontraditional factors, particularly extra-articular manifestations and functional disability, were associated with arterial vascular events. Awareness of these associations may help to implement clinical strategies in this group of rheumatoid arthritis patients at risk of arterial vascular events.

## Introduction

Rheumatoid arthritis (RA) is a chronic autoimmune disease characterized by a symmetric inflammatory arthritis and the presence of autoantibodies, particularly rheumatoid factor (RF) and antibodies to cyclic citrullinated peptides (CCPs).^1^ The prevalence is estimated to be 0.5%–1.0%.^2^ Women are affected 2–3 times more frequently than men, and the onset of disease usually occurs between 40 and 60 years of age. The pathogenesis of RA remains unknown, but both genetic and environmental factors have been implicated.^3^ RA can also present with extra-articular manifestations involving the cutaneous, ocular, respiratory, hematological, neurological, and cardiovascular systems. Management of patients with RA requires an interdisciplinary approach that involves both functional and psychosocial interactions.^4^ Early diagnosis and treatment with conventional and/or biological disease-modifying anti-rheumatic drugs (DMARDs) have improved the long-term outcome of RA.

Cardiovascular disease has a major impact in the morbidity and mortality of patients with RA.^5–7^ Both traditional and nontraditional risk factors have been found to be associated with these events in RA patients. Traditional factors include cigarette smoking, dyslipidemia, diabetes mellitus (DM), and metabolic syndrome. On the contrary, the inflammatory process intrinsic to RA seems to also contribute to the occurrence of arterial vascular events. Inflammatory states may increase the risk of vulnerability and rupture of atherosclerotic plaques.^8^ Furthermore, some studies have linked the incidence of vascular events with high levels of inflammatory biomarkers at baseline.^9–11^ Consistent with this notion, some studies have shown that early treatment of RA can reduce the risk of developing these events.^12^ Traditional and nontraditional risk factors for arterial vascular events vary among different ethnic groups.^13^ However, limited studies have been conducted in Hispanics from Latin America and the United States. Thus, the aim of this observational study was to determine the factors associated with the occurrence of arterial vascular events in a cohort of Hispanics from Puerto Rico with RA.

## Methods

### Patient population

A cross-sectional study was performed in a cohort of 405 patients with RA. Consecutive RA patients at the University of Puerto Rico Medical Sciences Campus (UPR-MSC) at San Juan, Puerto Rico; the University of Puerto Rico Hospital at Carolina, Puerto Rico; and four private rheumatology practices located in San Juan, Puerto Rico, were followed from February 2007 to June 2015. Data for this cross-sectional study were gathered from January 2015 to June 2015. Patients who fulfilled the 1987 revised criteria of the American College of Rheumatology (ACR) for the classification of RA,^14^ had Puerto Rican ethnicity (self and four grandparents), and were ⩾21 years of age (age of majority in Puerto Rico) were included in the study. Those who did not meet RA classification criteria, had non-Puerto Rican ethnicity, or were younger than 21 years of age at enrollment were excluded. This study was approved by the UPR-MSC Institutional Review Board (Protocol No. A4560107). Signed consent was waived because this research presented no more than minimal risk of harm to subjects and involved no procedures for which written consent is normally required. The clinical investigators (R.M.F.J., M.A.-A., L.D.C., N.R., G.R., and L.M.V.) of this study collected data from patients’ medical records of their own university- and/or community-based rheumatology practices. Each patient studied received a unique identifier number, and clinical information gathered was analyzed using this study number only. Data from study forms were entered in an electronic controlled-access database. Only researchers participating in this study had access to this database. The electronic database did not include any identifying information.

RA patients had their routine visits with their rheumatologists at 3-month intervals. Additional visits were scheduled as required by worsening of disease activity or the occurrence of new disease complications. At each routine visit, laboratory testing such as complete blood cell count, serum chemistries, urine analysis, erythrocyte sedimentation rate (ESR), C-reactive protein (CRP), and lipid panel were routinely ordered. ESR was measured using Westergren method. RF and anti-CCP antibodies were obtained at diagnosis. Clinical history, examination, and recording of clinical data and investigations were standardized. At each visit, a structured RA clinical form was completed for each patient to gather data about demographic features, lifestyle behaviors, RA clinical manifestations, disease activity, comorbidities, hospitalizations, joint surgeries, pharmacological treatment, and functional status. This form was created by the UPR-MSC Rheumatology Division to gather clinical information uniformly, allowing the assessment of RA outcomes measures at each visit. Also, medical records of RA patients were reviewed to gather further information of clinical manifestations, comorbidities, hospitalizations, and pharmacological therapy.

### Variables

For the analysis, the cohort was divided in two groups based on the occurrence of a vascular event after the onset of RA. Arterial vascular events were defined as the occurrence of angina pectoris, myocardial infarction, and reported vascular procedures for coronary artery disease, stroke, or peripheral artery disease. Data of arterial vascular events were obtained from patients themselves, and their hospital and subspecialty-practice medical records. Data included relevant diagnostic or interventional investigations such as coronary angiography, exercise testing, imaging studies, and vascular examinations. Four RA patients had arterial vascular events prior to RA diagnosis. Since only incident arterial vascular events were studied, these patients were excluded from the analyses.

Demographic characteristics, lifestyle behaviors, RA clinical manifestations, disease activity, functional status, and pharmacological profile were studied. Demographic features examined included age at study visit, sex, and disease duration. The latter was defined as the time interval between the onset of RA and the study visit. Lifestyle behaviors were reported by patients at study visit and included alcohol consumption, cigarette smoking, and exercise. Alcohol consumption was defined as ⩾1 drink (14.0 g or 0.6 ounces of pure alcohol) per day for women and ⩾2 drinks per day for men. Exercise was defined as regular participation in physical activity (aerobic exercise and/or muscle strength training) as part of a personal fitness plan—at least three times per week for at least 30 min per session.

Cumulative (at any time) RA manifestations, including joint deformities, radiographic joint damage (erosions and/or joint space narrowing), extra-articular manifestations (subcutaneous nodules, ocular, pulmonary, cardiac, and neurological), RF, anti-CCP antibodies, elevation of ESR and CRP, and highest recorded ESR, were determined. Positive tests of RF and anti-CCP antibodies referred to values that were higher than the upper limit of normal for the assay. Disease activity and patient’s pain assessment were assessed using the Disease Activity Score 28 (DAS-28) and by visual analogue scale, respectively.^15,16^ Functional status was ascertained using the Health Assessment Questionnaire (HAQ) Disability Index (Supplemental Material).^17,18^ The HAQ is a validated instrument created to assess the functional status in patients with arthritis. The HAQ has been translated and validated in many languages, including Spanish, which is the official language in Puerto Rico.^18^ The number of RA exacerbations, joint surgeries, and number of hospitalizations attributed to RA complications were assessed. RA exacerbation was defined as the onset of new joint, organ, and/or system involvement, or worsening of disease that requires modification of therapy.

The following cumulative comorbidities were examined: overweight/obesity (by body mass index, BMI), arterial hypertension (⩾130 mm Hg systolic blood pressure or ⩾85 mm Hg diastolic blood pressure or drug treatment for patients with hypertension), type 2 DM, dyslipidemia, metabolic syndrome, peripheral venous disease, chronic obstructive pulmonary disease, depression, malignancy, Sjögren’s syndrome, osteoarthritis, chronic low back pain (back pain lasting for more than 3 months), and fibromyalgia syndrome. Dyslipidemia was defined as elevated total cholesterol ⩾200 mg/dl or low-density lipoprotein cholesterol ⩾100 mg/dl or drug treatment to reduce cholesterol levels, and/or elevated triglycerides ⩾150 mg/dl or drug treatment for hypertriglyceridemia. Metabolic syndrome was ascertained using the Adult Treatment Panel III (ATP III) classification modified by the American Heart Association/National Heart, Lung, and Blood Institute.^19^ Comorbidities were selected based on previous studies showing the association of these conditions with arterial vascular events.

The cumulative use of non-steroidal anti-inflammatory drugs (NSAIDs), corticosteroids, and conventional synthetic and biological DMARDs was determined.

### Statistical analysis

In total, 405 patients evaluated in two groups (43 with incident arterial vascular events and 362 patients without arterial vascular events) with an alpha level of 0.05. Variables with group proportions that differed between 20% and 30% detected differences between groups with a 72% to 98% statistical power.

Distribution of all study variables was determined through descriptive statistics (percentages, mean, and standard deviations). Chi-square test and Fisher’s exact test were used, as appropriate, to assess differences between the presence and absence of arterial vascular events for categorical variables (e.g. gender, alcohol consumption, BMI categories, type 2 diabetes, and metabolic syndrome). Student’s t test or Mann–Whitney test, as appropriate, were performed to determine the differences between the presence or absence of arterial vascular events for continuous variables (e.g. disease duration, number of hospitalizations, and patient’s functional status). Bartlett’s and Shapiro–Wilk tests were used to evaluate assumptions of homoscedasticity and normality, respectively, to determine the correct assessment for each of the continuous variables evaluated on the study. Multivariate logistic regression models were performed to estimate odds ratios (ORs), along with their 95% confidence intervals (CIs), for all variables that showed a statistically significant difference (p ⩽ 0.05) in the bivariate analyses. All models were adjusted by age and gender. Statistical analyses were performed using the Statistical Package STATA (StataCorp. 2015, Release 14, College Station, TX, USA).

## Results

The cohort consisted of 405 RA patients, of which 353 (87.2%) were females. The mean age of the study population at the study visit was 56.1 ± 13.9 years, and the mean disease duration was 15.0 ± 13.2 years. During the course of RA, 43 (10.6%) patients had arterial vascular events. Fifteen patients had cerebrovascular accidents, 12 had myocardial infarction, 9 had angina pectoris, 9 had peripheral arterial disease, and 8 underwent coronary artery bypass graft. Ten patients had more than one event. Demographic features and health-related behaviors in RA patients with and without cardiovascular events are depicted in Table 1. Arterial vascular events were more frequent in older (63.0 ± 11.1 vs 55.2 ± 14.0 years, p < 0.001) and in male patients (19.2% vs 9.3%, p = 0.031). Regarding lifestyle behaviors, no differences were found for alcohol consumption, cigarette smoking, or exercise.

**Table 1.** Socio-demographic features and lifestyle behaviors in RA patients with and without arterial vascular events.

Features	Vascular events (n = 43)	No vascular events (n = 362)	p value
Age, years, mean (SD)	63.0 (11.1)	55.2 (14.0)	<0.001
Gender, %			
Female	9.3	90.7	0.031
Male	19.2	80.8	
Duration of RA, years, mean (SD)	14.9 (10.9)	15.0 (13.5)	0.984
Alcohol consumption, %	2.3	5.0	0.436
Smoking, %	9.3	6.6	0.514
Exercise, %	11.9	18.5	0.291

RA: rheumatoid arthritis.

Clinical manifestations, disease activity, functional status, and clinical outcomes are described in Table 2. Patients who developed an arterial vascular event were more likely to have extra-articular manifestations (69.8% vs 48.9%, p = 0.010), pulmonary manifestations (18.6% vs 6.4%, p = 0.004), and higher DAS-28 score (4.2 ± 1.8 vs 3.6 ± 1.4, p = 0.044), HAQ score (1.4 ± 0.9 vs 1.1 ± 0.8, p = 0.017), and number of hospitalizations (0.4 ± 0.9 vs 0.1 ± 0.5, p = 0.027) than those without arterial vascular events. No significant differences were found for joint deformities, radiographic changes, RF, anti-CCP antibodies, ESR, CRP, other extra-articular manifestations (subcutaneous nodules, ocular, cardiac, neurological), patient’s pain assessment, exacerbations, or joint surgeries.

**Table 2.** Clinical manifestations, disease activity, functional status, and clinical outcomes in RA patients with and without arterial vascular events.

Clinical manifestations	Vascular events (n = 43)	No vascular events (n = 362)	p value
Joint deformities, %	53.5	48.6	0.546
Radiographic changes, %	77.4	65.2	0.170
Positive rheumatoid factor, %	73.7	64.9	0.279
Positive anti-CCP, %	64.3	73.3	0.467
Elevated ESR, %	83.7	87.7	0.458
Highest recorded ESR, mm/h, mean (SD)	74.1 (25.9)	67.2 (27.9)	0.083
Elevated CRP, %	80.0	66.7	0.223
Extra-articular manifestations, %	69.8	48.9	0.010
Subcutaneous nodules	34.9	25.4	0.183
Ocular	46.5	34.5	0.121
Pulmonary	18.6	6.4	0.004
Cardiac	2.3	0.3	0.070
Neurological	27.9	18.5	0.141
DAS-28, mean (SD)	4.2 (1.8)	3.6 (1.4)	0.044
Patient’s pain assessment, mean (SD)	48.9 (29.6)	45.9 (30.0)	0.530
Patient’s functional status, HAQ, mean (SD)	1.4 (0.9)	1.1 (0.8)	0.017
RA exacerbations, mean (SD)	2.4 (2.2)	2.1 (3.1)	0.553
Joint replacement surgery, %	11.6	19.7	0.202
Hospitalizations, mean (SD)	0.4 (0.9)	0.1 (0.5)	0.027

RA: rheumatoid arthritis; anti-CCP: anti-cyclic citrullinated peptide; ESR: erythrocyte sedimentation rate; CRP: C-reactive protein; DAS-28: Disease Activity Measure 28; HAQ: Health Assessment Questionnaire.

Selected comorbidities are shown in Table 3. Patients with arterial vascular events presented with higher frequency of arterial hypertension (83.7% vs 51.7%, p < 0.001), type 2 DM (25.6% vs 13.8%, p = 0.041), dyslipidemia (81.0% vs 45.3%, p < 0.001), metabolic syndrome (44.2% vs 24.3%, p = 0.005), peripheral venous disease (11.6% vs 3.9%, p = 0.040), and chronic low back pain (30.2% vs 15.2%, p = 0.013) than those without these events.

**Table 3.** Selected comorbid conditions in RA patients with and without arterial vascular events.

Comorbid conditions	Vascular events (n = 43) (%)	No vascular events (n = 362) (%)	p value
Body mass index categories			
Normal (18.5–24.9)	30.9	34.1	0.513
Overweight (25–29.9)	31.0	36.4	
Obese (⩾30)	38.1	29.5	
Non obese (<30)	61.9	70.5	0.252
Obese (⩾30)	38.1	29.5	
High blood pressure	83.7	51.7	<0.001
Type 2 diabetes mellitus	25.6	13.8	0.041
Dyslipidemia	81.0	45.3	<0.001
Metabolic syndrome	44.2	24.3	0.005
Peripheral venous disease	11.6	3.9	0.040
Chronic obstructive pulmonary disease	14.0	6.9	0.100
Depression	18.6	12.7	0.282
Malignancy	9.3	3.6	0.077
Sjögren’s syndrome	11.6	8.6	0.504
Osteoarthritis	69.8	60.8	0.251
Chronic low back pain	30.2	15.2	0.013
Fibromyalgia syndrome	11.6	6.1	0.168

RA: rheumatoid arthritis.

No significant differences between study groups were observed for any of the RA treatment categories (NSAIDs, corticosteroids, conventional synthetic and biological DMARDs) (Table 4). Furthermore, no differences were observed specifically for methotrexate, hydroxychloroquine, or tumor necrosis factor (TNF) inhibitors.

**Table 4.** Pharmacological therapies in RA patients with and without arterial vascular events.

Pharmacological therapies	Vascular events (n = 43) (%)	No vascular events (n = 362) (%)	p value
Non-steroidal anti-inflammatory drugs	79.1	86.5	0.191
Corticosteroids	88.4	75.1	0.052
Disease modifying anti-rheumatic drugs	93.0	93.9	0.817
Conventional synthetic			
Hydroxychloroquine	60.5	52.2	0.305
Methotrexate	86.1	83.4	0.660
Sulfasalazine	20.9	15.2	0.330
Biological agents	46.5	49.7	0.690
Tumor necrosis factor blockers	46.5	48.6	0.794
Abatacept	7.0	6.3	0.748
Rituximab	0.0	0.6	0.999
Tocilizumab	0.0	0.3	0.999

RA: rheumatoid arthritis.

In the multivariate analysis adjusted for age and gender, arterial vascular events were associated with extra-articular manifestations (OR = 2.08, 95% CI = 1.03–4.20), worse functional status (OR = 2.52, 95% CI = 1.25–5.11), higher number of hospitalizations (OR = 3.44, 95% CI = 1.54–7.69), arterial hypertension (OR = 3.43, 95% CI = 1.40–8.41), dyslipidemia (OR = 4.48, 95% CI = 2.00–10.04), and metabolic syndrome (OR = 1.98, 95% = 1.02–3.87). These results are depicted in Table 5. Disease activity (per DAS 28), DM, and peripheral venous disease did not retain significance.

**Table 5.** Logistic regression analysis for factors associated with arterial vascular events among RA patients.

Features	Crude OR (95% CI)	Adjusted OR^a^ (95% CI)
Gender^b^	2.31 (1.06–5.02)	2.14 (0.97–4.73)
Age	1.05 (1.02–1.07)	1.04 (1.01–1.07)
Extra-articular manifestations	2.41 (1.22–4.77)	2.08 (1.03–4.20)
Pulmonary extra-articular manifestations	3.37 (1.40–8.10)	2.45 (0.98–6.11)
Disease activity, DAS-28	1.26 (1.03–1.54)	1.49 (0.77–2.90)
Patient’s functional status, HAQ	2.39 (1.20–4.75)	2.52 (1.25–5.11)
Hospitalizations	3.67 (1.69–7.99)	3.44 (1.54–7.69)
High blood pressure	4.81 (2.05–11.10)	3.43 (1.40–8.41)
Type 2 diabetes mellitus	2.15 (1.02–4.53)	1.62 (0.75–3.52)
Dyslipidemia	5.13 (2.31–11.40)	4.48 (2.00–10.04)
Metabolic syndrome	2.46 (1.29–4.71)	1.98 (1.02–3.87)
Peripheral venous disease	3.27 (1.12–9.58)	2.93 (0.96–8.97)

RA: rheumatoid arthritis; OR: odds ratio; CI: confidence interval; DAS-28: Disease Activity Measure 28; HAQ: Health Assessment Questionnaire.

a OR were adjusted by gender and age; gender was adjusted for age only; age was adjusted for gender only.

b Females were used as the reference category for gender in the crude and adjusted logistic regression models.

## Discussion

Cardiovascular disease has been found to be a burden for patients with RA, contributing to an increased morbidity and mortality of this population.^20,21^ Traditional factors and the inflammatory characteristics inherent of the disease have an impact on the development of arterial vascular events of these patients. However, the prevalence and clinical correlates of these events in RA patients vary among different ethnic populations. Herein, we assessed the factors associated with arterial vascular events in Hispanics from Puerto Rico with RA and found that both traditional (arterial hypertension, dyslipidemia, and metabolic syndrome) and nontraditional (extra-articular manifestations and functional impairment) factors to be significantly associated with incident arterial vascular events.

In our study, 10.6% of RA patients had arterial vascular events during the course of disease. This prevalence is slightly higher than that reported in the multicentric study QUEST-RA (Quantitative Patient Questionnaires in Standard Monitoring of Patients with Rheumatoid Arthritis) in which the overall prevalence of cardiovascular events was 9.3%.^22^ In that study, populations that are relevant to ours in terms of genetic background or geographical proximity were studied. For example, Hispanics from Argentina had a relatively low prevalence of cardiovascular events (3.7%) compared to 11.6% in the United States and 10.0% in Spain.^22^ In a case control study, using data from the CARMA (Cardiovascular in Rheumatology) project, RA patients from Spain had a higher prevalence of cardiovascular events (10.5%) when compared to patients with ankylosing spondylitis (AS) and psoriatic arthritis (PsA).^23^ In another multinational RA cohort, CORRONA, arterial vascular events were more commonly seen in Eastern European regions (21.3%), followed by Latin America (8.5%) and the United States (8.5%).^13^

We found that arterial vascular events were more frequent in men than in women. Reports on the influence of gender and the risk of cardiovascular events are controversial. Nonetheless, in agreement with this observation, multiple reports suggest an increased prevalence of cardiovascular events in men with RA,^23,24^ even after adjusting for cardiovascular risk factors.^25^ Interestingly, in a group of RA patients with low disease activity and no history of cardiovascular disease, men had a significantly higher risk of cardiovascular-related mortality and atherosclerosis when compared to females.^26^ The differences observed between our study and others could be related to diversity in genetic and socioeconomic factors, access to health care, comorbidities, and length of follow-up, among others.

Traditional factors that confer risk to cardiovascular events in RA patients are not quite different from those seen for the general population. However, their impact may vary among different ethnic populations. Our data, as well as the literature, confirm that hypertension (83.7%) and hyperlipidemia (81.0%) are associated with arterial vascular events in RA patients.^25,27^ Other factors that have been consistently found to be linked with cardiovascular events are tobacco smoking and DM.^25,28^ In the bivariate analysis of our study, type 2 DM was associated with arterial vascular events, but significance was not retained in the multivariate analysis. Also, in an international cohort, smoking and hypertension were associated with an increased overall risk among both sexes.^25^ Interestingly, in the CORRONA study, the prevalence of hypertension (39.4%), dyslipidemia (18.6%), and DM (8.4%) in Latin Americans with cardiovascular events appears to be much lower than that reported for our population.^13^ This observation was also seen in the CARMA cohort, where the prevalence of hypertension (65.1%), dyslipidemia (53.5%), and DM (23.3%) was lower in Spaniards with cardiovascular events.^23^ These discrepancies could be related to the fact that RA patients were approximately 11 years younger in the CORRONA study and 14 years younger in the CARMA study in comparison to ours. Also, the prevalence of obesity (BMI >30) was greater in our study group (38.1%) as compared to Latin Americans in the CORRONA study (25.5%) and Spaniards in the CARMA study (23.3%).

Obesity, as a traditional factor, has been described as a contributor to a low-grade inflammation state given the increased production of cytokines such as IL-6 and adinopectin.^8^ Sixty-nine percent of RA patients with cardiovascular events in our cohort were classified as overweight or obese per BMI. In our study, both in the bivariate and multivariate analyses, metabolic syndrome was associated with cardiovascular events. Studies have shown a link between RA and metabolic syndrome, reporting an increased frequency of metabolic syndrome in RA patients. In a meta-analysis, a worldwide prevalence of metabolic syndrome of 30.7% was reported for RA patients with a significant association between RA and the risk of metabolic syndrome.^29^

Previous studies have highlighted the relevance of nontraditional factors as a major cause to develop arterial vascular events in RA patients. Noteworthy, some studies have demonstrated that the risk of developing carotid plaque instability and arterial stiffness in RA patients is comparable to that seen in diabetic patients.^30^ In our study, extra-articular manifestations and functional impairment were associated with arterial vascular events. These findings are in agreement with other studies. The most common extra-articular manifestations in RA patients presenting with cardiovascular events are subcutaneous nodules and lung disease.^31–33^ In our cohort, pulmonary manifestations were associated with vascular events, and among them, nearly 70% of patients had interstitial lung disease. The relationship with pulmonary involvement is not surprising as patients with idiopathic pulmonary fibrosis have a two-time risk of developing cardiovascular disease, including acute coronary syndrome and deep venous thrombosis as compared to the general population.^34,35^ In terms of functional impairment, few studies have reported the association with arterial vascular events. Farragher et al.^36^ reported an increased mortality rate related to cardiovascular disease in RA patients presenting with higher HAQ score. Similarly, in the CARMA project, when compared to AS and PsA, the RA group presented a significant association between higher HAQ scores and increased risk of cardiovascular events.^23^ Finally, we found that patients with arterial vascular events had more hospitalizations than those without these events. This finding was expected because most of these patients required hospitalizations to manage their vascular diseases and related complications.

It has been postulated that the systemic release of pro-inflammatory cytokines from RA synovial tissue could enhance the inflammatory process, consequently accelerating the atherosclerotic process.^11,37^ Some investigators have reported a protective effect in patients with lower disease activity (DAS-28 score of <3.2), and worst outcomes in those with higher disease activity in relation to cardiovascular disease.^38,39^ In the bivariate analysis, but not in the multivariate analysis, disease activity per DAS-28 was higher in patients with arterial vascular events. It must be noted that we measured DAS-28 at study visit and not throughout the course of disease; thus, it may not reflect disease activity at earlier stages, before the occurrence of arterial vascular events.

Unlike other studies in RA, our data did not confirm any association between the RA treatments and cardiovascular events. A protective effect has been reported with the use of hydroxychloroquine ^40^ and other DMARDs when initiated within the first 3 months of RA diagnosis.^12^ Regarding treatment with corticosteroids, many studies suggest an increased risk of cardiovascular events in patients with long-term use of corticosteroids.^41^ Other authors have also reported a favorable impact of TNF antagonists in reducing the effects of cardiovascular events when initiated at early stages of the disease.^22,41,42^

This study has some limitations. First, this study has the limitations inherent to its design. Cross-sectional studies have potential biases in assessing a causal relationship because it may not be possible to determine whether the potential exposure preceded or not the outcome. Conversely, this type of observational research may have an important role in providing information required to improve health care. Second, arterial vascular events were grouped into one variable as the number of individual conditions was relatively small to warrant statistical analysis. Third, we could not clearly examine the association with inflammatory markers such as ESR and CRP, as longitudinal data of these tests were not available in our database. Previous studies have shown that CRP is an independent factor for cardiovascular events in patients with RA ^43^. Fourth, data regarding the cumulative corticosteroid dose or treatment duration of DMARDs or biological agents were not available to explore the harmful or protective role of these medications. Finally, stratification of various risk factors like exercise, smoking, or alcohol was not determined. This limitation was also the case for socioeconomic features. Nevertheless, our study presents comprehensive data about clinical correlates of arterial vascular events in a cohort of Hispanics with RA followed for a prolonged period.

## Conclusion

In summary, arterial hypertension, dyslipidemia, metabolic syndrome, extra-articular manifestations, and poor functional status were associated with arterial vascular events in our Hispanic RA cohort. This study further supports the notion that both traditional and nontraditional factors play an important role in the occurrence of arterial vascular events in patients with RA. Our data contribute to the few studies that have been conducted in Hispanics with RA.

## Supplemental Material

SOM-19-0429.R2_-_Health_Assessment_Questionnaire_-_English_version – Supplemental material for Incident arterial vascular events in a cohort of Puerto Ricans with rheumatoid arthritis Click here for additional data file. Supplemental material, SOM-19-0429.R2_-_Health_Assessment_Questionnaire_-_English_version for Incident arterial vascular events in a cohort of Puerto Ricans with rheumatoid arthritis by Ariana González-Meléndez, Ruth M Fred-Jiménez, Mariangelí Arroyo-Ávila, Leyda Díaz-Correa, Naydi Pérez-Ríos, Noelia Rodríguez, Grissel Ríos and Luis M Vilá in SAGE Open Medicine

SOM-19-0429.R2_-_Health_Assessment_Questionnaire_-_Spanish_version – Supplemental material for Incident arterial vascular events in a cohort of Puerto Ricans with rheumatoid arthritis Click here for additional data file. Supplemental material, SOM-19-0429.R2_-_Health_Assessment_Questionnaire_-_Spanish_version for Incident arterial vascular events in a cohort of Puerto Ricans with rheumatoid arthritis by Ariana González-Meléndez, Ruth M Fred-Jiménez, Mariangelí Arroyo-Ávila, Leyda Díaz-Correa, Naydi Pérez-Ríos, Noelia Rodríguez, Grissel Ríos and Luis M Vilá in SAGE Open Medicine
