# Supplementary material for: Incident arterial vascular events in a cohort of Puerto Ricans with rheumatoid arthritis
Source: SAGE Open Med. 2020 Sep 15;8:2050312120958844. doi: 10.1177/2050312120958844 (PMC7495931; doi:10.1177/2050312120958844)
Supplement: SOM-19-0429.R2_-_Health_Assessment_Questionnaire_-_English_version – Supplemental material for Incident arterial vascular events in a cohort of Puerto Ricans with rheumatoid arthritis [file SOM-19-0429.R2_-_Health_Assessment_Questionnaire_-_English_version.pdf]

Health Assessment Questionnaire (HAQ) Disability Index - English version

Please place an "x" in the box which best describes your abilities OVER THE PAST WEEK:	**Without any difficulty**	**With some difficulty**	**With much difficulty**	**Unable to do**
**DRESSING & GROOMING, Are you able to:**				
Dress yourself, including shoelaces and buttons?				
Shampoo your hair?				
**ARISING, Are you able to:**				
Stand up from a straight chair?				
Get in and out of bed?				
**EATING, Are you able to:**				
Cut your own meat?				
Lift a full cup or glass to your mouth?				
Open a new milk carton?				
**WALKING, Are you able to:**				
Walk outdoors on flat ground?				
Climb up five steps?				
**HYGIENE, Are you able to:**				
Wash and dry your body?				
Get on and off the toilet?				
**REACH, Are you able to:**				
Reach and get down a 5 pound object (such as a bag of sugar) from above your head?				
Bend down to pick up clothing from the floor?				
**GRIP, Are you able to:**				
Open car doors?				
Open previously opened jars?				
Turn faucets on and off?				
**ACTIVITIES, Are you able to:**				
Run errands and shop?				
Get in and out of a car?				
Do chores such as vacuuming or yard work?				
