# Supplementary material for: Incident arterial vascular events in a cohort of Puerto Ricans with rheumatoid arthritis
Source: SAGE Open Med. 2020 Sep 15;8:2050312120958844. doi: 10.1177/2050312120958844 (PMC7495931; doi:10.1177/2050312120958844)
Supplement: SOM-19-0429.R2_-_Health_Assessment_Questionnaire_-_Spanish_version – Supplemental material for Incident arterial vascular events in a cohort of Puerto Ricans with rheumatoid arthritis [file SOM-19-0429.R2_-_Health_Assessment_Questionnaire_-_Spanish_version.pdf]

Health Assessment Questionnaire (HAQ) Disability Index - Spanish version

Por favor, marque la respuesta que mejor describa sus capacidades habituales durante la última semana.	Sin NINGUNA dificultad	Con ALGUNA dificultad	Con MUCHA dificultad	No puedo hacerlo
**VESTIRSE Y ARREGLARSE - ¿Puede:**				
vestirse, incluyendo cordones de los zapatos y botones?				
ponerse champú en el cabello?				
**LEVANTARSE - ¿Puede:**				
levantarse de una silla recto?				
acostarse y levantarse de la cama?				
**COMER - ¿Puede:**				
cortar la carne?				
levantar una taza o vaso llenos hasta su boca?				
abrir un cartón nuevo de leche?				
**CAMINAR - ¿Puede:**				
caminar afuera en terreno plano?				
subir cinco escalones?				
**HIGIENE - ¿Puede:**				
bañarse y secarse el cuerpo?				
sentarse y levantarse del inodoro?				
**ALCANZAR - ¿Puede:**				
alcanzar y bajar un objeto de 5 libras (como una bolsa de azúcar) por encima de su cabeza?				
inclinarse y recoger la ropa?				
**AGARRAR- ¿Puede:**				
abrir las puertas de autos?				
abrir frascos abiertos previamente?				
abrir y cerrar llaves de agua?				
**ACTIVIDADES - ¿Puede:**				
hacer los mandados e ir de compras?				
entrar y salir de un auto?				
hacer labores como aspirar o trabajar en el patio?				
